# Pulmonary biomarkers in COPD exacerbations: a systematic review

**DOI:** 10.1186/1465-9921-14-111

**Published:** 2013-10-21

**Authors:** Angela Koutsokera, Konstantinos Kostikas, Laurent P Nicod, Jean-William Fitting

**Affiliations:** 1 Department of Respiratory Medicine, University Hospital of Lausanne, Lausanne, Switzerland; 2 2nd Respiratory Medicine Department, University of Athens Medical School, Attikon Hospital, Athens, Greece; 3 Service de Pneumologie, Centre Hospitalier Universitaire Vaudois, Rue du Bugnon 46, 1011 Lausanne, Switzerland

**Keywords:** COPD (chronic obstructive pulmonary disease), Exacerbations of COPD, Biomarkers, Airway inflammation, Cytokines, Spontaneous sputum, Induced sputum, Exhaled breath condensate, Fractional exhaled nitric oxide, Bronchoalveolar lavage

## Abstract

Exacerbations of COPD (ECOPD) represent a major burden for patients and health care systems. Innovative sampling techniques have led to the identification of several pulmonary biomarkers. Although some molecules are promising, their usefulness in clinical practice is not yet established. Medline and Highwire databases were used to identify studies evaluating pulmonary sampled biomarkers in ECOPD. We combined 3 terms for ECOPD, 3 for biomarkers and 6 for the sampling method. Seventy-nine studies were considered eligible for inclusion in the review and were analyzed further. Pulmonary biomarkers sampled with non-invasive, semi-invasive and invasive methods were evaluated for their potential to illustrate the disease’s clinical course, to correlate to clinical variables and to predict clinical outcomes, ECOPD etiology and response to treatment. According to published data several pulmonary biomarkers assessed in ECOPD have the potential to illustrate the natural history of disease through the modification of their levels. Among the clinically relevant molecules, those that have been studied the most and appear to be promising are spontaneous and induced sputum biomarkers for reflecting clinical severity and symptomatic recovery, as well as for directing towards an etiological diagnosis. Current evidence on the clinical usefulness of exhaled breath condensate and bronchoalveolar lavage biomarkers in ECOPD is limited. In conclusion, pulmonary biomarkers have the potential to provide information on the mechanisms underlying ECOPD, and several correlate with clinical variables and outcomes. However, on the basis of published evidence, no single molecule is adequately validated for wide clinical use. Clinical trials that incorporate biomarkers in decisional algorithms are required.

## Background

The natural history of chronic obstructive pulmonary disease (COPD) is marked by episodes of deterioration, called exacerbations of COPD (ECOPD) which lead to increased morbidity and mortality [1]. An ERS/ATS Task Force published in 2008 has described ECOPD as one of the clinical outcomes of COPD that should be used for the assessment of patients and for defining the impact of treatment interventions [2]. In addition to the functional and imaging markers, increasing evidence suggests that sampling, either local or systemic, of biological molecules known as biomarkers can provide an insight in the pathophysiological mechanisms of ECOPD [2,3]. Sampling methods elaborated during the last decades offer an innovative basis for the identification of pulmonary biomarkers. These techniques may be totally *noninvasive* [e.g. exhaled air, exhaled breath condensate (EBC), spontaneous sputum (SS)], *semi-invasive* (e.g. induced sputum (IS), nasal wash, large airways’ secretions) or *invasive* [e.g. bronchoalveolar lavage (BAL), lung biopsies]. Although readily implemented in the research laboratory, their wide application has long been hampered by the lack of standardization and the absence of reference values, issues that are being increasingly addressed in the literature [4,5]. The aim of this systematic review is to provide an overview of the pulmonary sampled biomarkers studied in the context of ECOPD and to highlight their associations with clinical variables in an attempt to illustrate the potential clinical implications of current evidence.

## Methodology and definitions

**Figure 1:**
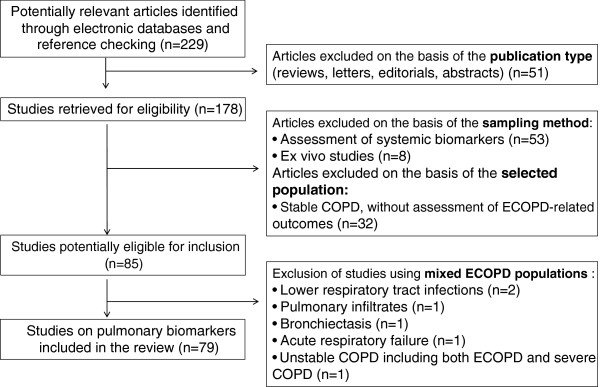
Flow chart diagram of the search strategy and study selection.

A search for articles, published in the English language until April 2013, was conducted using Medline and Highwire databases as well as the reference lists of retrieved publications. Studies were considered pertinent if they evaluated non-systemic, pulmonary sampled biological molecules in the context of ECOPD. Research included – but was not limited to - 3 keywords for ECOPD (COPD exacerbation, COPD deterioration, acute COPD), 3 for biomarkers (biomarkers, cytokines, oxidative stress) and 6 for the sampling methods (exhaled, EBC, BAL, IS, SP, lung biopsies). Abstracts or unpublished reports were not included in the current review, whereas data obtained from mixed populations were excluded from further analysis. Data on cellular, functional or imaging parameters that may be used as markers of disease were not included in the present review. Biomarkers sampled with *non-invasive, semi-invasive or invasive methods* were evaluated for their potential: 1) to illustrate the natural history of ECOPD through the modification of their levels, 2) to correlate with clinical variables [e.g. symptoms, clinical severity, pulmonary function tests (PFTs) and arterial blood gas (ABG)] and 3) to serve as predictors of clinical outcomes (e.g. recovery, length of hospital stay and ECOPD frequency), ECOPD etiology and response to treatment. The methodology used was in accordance with the PRISMA guidelines [6]. A flow chart diagram of the search strategy and study selection is provided in Figure 1.

As described previously for systemic biomarkers in ECOPD [7] the following terminology was applied invariably:

•**‘Baseline’** refers to a time-point of clinical stability, before the development of ECOPD (used for longitudinal studies).

•**‘ECOPD onset’** refers to the first time-point at which ECOPD patients were assessed by the investigators.

•The terms ‘**stability or recovery’** were avoided and the specific time point of assessment was preferably used.

•**‘Stable COPD’** refers to cross-sectional studies comparing ECOPD patients with another group of COPD patients.

## Results and discussion

### Non-invasive sampling

#### Exhaled biomarkers

Breath analysis is considered to be a valuable non-invasive technique for sampling volatile biomarkers. With the exception of one study which assessed four volatile biomarkers simultaneously [8], fractional exhaled nitric oxide (FeNO) is the only exhaled biomarker studied in ECOPD (Table 1). Electronic nose, a recently developed omics technique that provides a ‘breathprint’ of exhaled volatile organic compounds, has not been studied yet during ECOPD [9].

**Table 1:** Assessment of FeNO in ECOPD

**Ref.**	**Course:**	**At ECOPD onset**		**Course:**	**Comment**
	**From Baseline to**	**FeNO levels**	**Comparisons**	**After ECOPD**	
	**ECOPD Onset**	**(ppb)**		**onset**	
Agusti et al. [10]		€41.0 ± 5.1, † 5.7-76.5	ECOPD > controls	↓	GCS. ↓ by M1-2 or by M6-8
Al-Ali et al. [11]		£10.3 (2.7-34)	↔ECOPD, controls, pneumonia		
			↔ECOPD smokers, ex smokers		
Antus et al. [12]		¥25.3 (21.2–30.1)		↓	GCS. ↓ by discharge
Antus et al. [13]		¥23.8 (19.4–29.7)	↔ECOPD smokers, ex smokers	↓	↓ by discharge
Bhowmik et al. [14]	↑*	#7.40 (4.80–9.60)		↓*	Exact time points N/R
Cosio et al. [15]		N/R		↓	GCS. ↓ by M3
Kersul et al. [16]		N/R	N/R	↓	GCS. ↓ by M3 after discharge
Lazar et al. [17]		‡10 (7)	↔ECOPD, controls (non-smokers, smokers)	N/R	Time points: admission, discharge
Papi et al. [18]		€15.18 ±1.85		↓	GCS. ↓ by W8-10

The columns concerning the course of FeNO refer to longitudinal studies (paired samples) and the column concerning comparisons at ECOPD onset refers to cross-sectional studies (unpaired samples).

*Abbreviations*: *D* day after ECOPD onset, *FeNO* fraction of exhaled nitric oxide, *GCS* administration of systemic glucocorticosteroids, *M* months after ECOPD onset, *N/R* not reported, *ppb* parts per billion, *W* weeks after ECOPD onset.

**Symbols:** ↔: no difference, ↑: increase, ↓: decrease, € mean ± SEM, † range, £ median (range), ¥ geometric mean (95% CI), # Median (interquartile range), ‡ mean (SD), *: stability samples obtained before and after the ECOPD.

Although, in stable COPD FeNO is derived predominantly from the periphery of the lung [19], in ECOPD it appears to be produced homogeneously in the central and peripheral airways [10]. FeNO at ECOPD onset is characterized by a wide range of concentrations and initially elevated levels as compared to controls have not been invariably documented [10,11]. This lack of data uniformity may be attributed to different study designs and confounding factors, such as smoking or inhaled corticosteroids (ICS), which are known to influence FeNO levels [20]. Despite the wide range of initial concentrations, most studies report a reduction of FeNO during follow-up [10,12-16,18].

FeNO was associated with the presence of a sore throat [14] and during viral ECOPD its levels correlated with sputum eosinophils [18]. Although, a correlation with PFTs has not been invariably demonstrated [10,13], in the study of Antus and coworkers, FeNO on admission correlated positively with post-treatment increases in FEV1, and the decrease of FeNO correlated with the increases in FEV1. In this study, FeNO on admission was a predictor of a significant post-treatment increase in FEV1 with an optimum cut-off point of 26.8 ppb. For this threshold sensitivity was 74%, specificity was 75% and positive and negative predictive values were 60 and 85% respectively [12]. ABG parameters did not correlate significantly with FeNO levels [13].

Concerning clinical outcomes, a weak inverse correlation has been reported between FeNO on admission and the length of hospital stay [12]. Finally, in regard to therapeutic interventions, intravenous glucocorticosteroids (GCS) failed to acutely reduce FeNO levels in ECOPD patients [10] whereas the co-administration of theophylline was not accompanied by an additional reduction of FeNO [15].

### Exhaled breath condensate

Exhaled breath is saturated with water vapor which can be condensed by cooling and used to sample a wide range of mediators. EBC samples the entire respiratory tract but newer techniques allow fractionated sampling and provide the ability to collect condensate from different parts of the respiratory tract [21,22]. EBC collection is a promising sampling method but several methodological issues hamper its clinical use [4,5]. In the context of ECOPD, detection rates of some of the sampled biomarkers, notably cytokines, have been particularly low [23,24] whereas for inflammatory mediators of a peptide nature, better detection rates have been achieved with albumin-coated sampling devices [23]. Studies comparing EBC, sputum or BAL in the context of ECOPD are scarce [24-26].

Only a few studies have focused on EBC biomarkers of ECOPD (Table 2) and so far no study has assessed the course of biomarkers between baseline and the onset of ECOPD. EBC ***pH***, one of the most validated EBC biomarkers, has been assessed only by one study which found no correlations with PFTs and ABG parameters [13]. ***NO-related products*** have not yet been evaluated in this context, whereas for hydrogen peroxide (***H***_***2***_***O***_***2***_) published data showed correlations with dyspnea but no correlations with the clinical status or the PFTs [27-29].

**Table 2:** Assessment of EBC biomarkers at ECOPD

**Biomarker**	**Ref.**	**ECOPD onset**	**Course:**	**Comment**
		**Comparisons**	**After ECOPD**	
			**onset**	
**AAT**	[30]	ECOPD > COPD, controls		
**ATP**	[17]	↔: ECOPD, smokers, non-smokers	↔	Time points: admission, discharge
**cysLTs**	[29]		↓	ICS with or without systemic GCS. Time points: onset, Visit 1 (D2-4), Visit 2 (2-4D post antibiotics), Visit 3 (21-28D post antibiotics). ↓ by visit 2.
**H**_**2**_**O**_**2**_	[27]	ECOPD > controls (smokers and non-smokers)	↓	GCS. ↓ by D7
	[28]		↓	GCS. ↓ by D3-4
	[29]		↓	ICS with or without GCS. Time points: onset, Visit 1 (D2-4), Visit 2 (2-4D post antibiotics), Visit 3 (21-28D post antibiotics). ↓ by visit 2.
**IL-1β**	[24]	ECOPD > controls, smokers, stable COPD		
**IL-6**	[31]	ECOPD > controls	↓	No GCS. ↓ by 2 W. Reduced after 6 M of mucolytic therapy
	[24]	ECOPD > controls, stable COPD, ECOPD _In GW_ > smokers		ICU or GW patients
**IL-8**	[24]	ECOPD > controls, smokers, stable COPD		ECOPD patients hospitalized either in the ICU or in GW
	[32]			Undetectable in most subjects
	[25]	ECOPD > nonsmokers, asymptomatic smokers, symptomatic smokers		Detected in 14% of healthy smokers, 20% of non-symptomatic smokers, 43% of symptomatic smokers
	[33]	ECOPD > asthma exacerbations, ↔: ECOPD, controls		GCS.
**IL-10**	[24]	ECOPD > controls, smokers, stable COPD		ICU or GW patients
**IL-12p70**	[24]	ECOPD > controls, smokers, stable COPD		ICU or GW patients
**IL-17**	[33]	↔: ECOPD, asthma exacerbations, controls		GCS.
**IFN-γ**	[23]	ECOPD < controls (p value N/R)		Improved detection when an albumin-coated collector was used
**8-isoprostane**	[31]	ECOPD > controls	↓	No GCS. ↓ by 2 W. Reduced after 6 M of mucolytic therapy
	[34]	ECOPD > controls	↓	No GCS. ↓ by 2 W. Reduced further within 2 M
	[25]	ECOPD > nonsmokers, asymptomatic smokers, Symptomatic smokers		
	[29]		↓	ICS with or without systemic GCS. Time points: onset, Visit 1 (D2-4), Visit 2 (2-4D post antibiotics), Visit 3 (21-28D post antibiotics). ↓ by visit 2.
**LTB4**	[34]	ECOPD > controls	↓	No GCS. ↓ by 2 W. Reduced further within 2 M
	[32]	↔: ECOPD, controls, stable COPD	↔	GCS. Time points: D5,14,30,60
	[29]		↓	ICS with or without systemic GCS. Time points: onset, Visit 1 (D2-4), Visit 2 (2-4D post antibiotics), Visit 3 (21-28D post antibiotics). ↓ by visit 3.
**MPO**	[23]	ECOPD > controls		For samples collected with an albumin-coated apparatus
**Ph**	[13]	↔: ECOPD, controls	↔	Time points: admission, discharge. CO2 standardization method
**PGE2**	[29]		↔	ICS with or without systemic GCS. Time points: onset, Visit 1 (D2-4), Visit 2 (2-4D post antibiotics), Visit 3 (21-28D post antibiotics).
**Protein**	[24]	↔: ECOPD, controls, smokers, stable COPD		
	[35]			
**SLPI**	[23]	ECOPD > controls		For samples collected with an albumin-coated apparatus
**TNF-α**	[24]	ECOPD > controls, smokers, stable COPD		ICU or GW patients
	[32]	↔: ECOPD on D5, controls, stable COPD	↑	GCS. Time points: D5, 14, 30, 60. Lowest levels on D5, D14 > D5 and D14 = D30 = D60
	[33]	↔: ECOPD, controls		GCS.

The column concerning the course of EBC biomarkers refers to longitudinal studies (paired samples) and the column concerning comparisons at ECOPD onset refers to cross-sectional studies (unpaired samples). No study has assessed the course of EBC biomarkers from baseline towards ECOPD onset.

*Abbreviations:AAT* alpha 1 antitrypsin, *ATP* adenosine triphosphate, *CysLTs* cysteinyl-leukotrienes, *D* day after ECOPD onset, *GCS* administration of systemic glucocorticosteroids, *GW* general ward, *H*_*2*_*O*_*2*_ hydrogen peroxide, *ICS* inhaled corticosteroids, *ICU* Intensive care unit, *IL* interleukin, *IFN-γ* interferon gamma, *LTB4* leukotriene B4, *M* months after ECOPD onset, min: minimum, *MPO* myeloperoxidase, *N/R* not reported, *PGE2* prostaglandin E2, *SLPI* secretory leukocyte protease inhibitor, *TNF-α* tumor necrosis factor alpha, *W* weeks after ECOPD onset.

**Symbols:** ↔: no difference, ↑: increase, ↓: decrease.

Isoprostanes and leukotrienes, are arachidonic acid metabolites that have also been measured in EBC samples. So far no double-blind placebo-controlled trial has focused on the effect of treatment interventions on EBC biomarkers. Definite conclusions cannot be drawn based on published evidence, but it has been shown that levels of ***8-isoprostane*** were elevated and fell significantly after treatment, decreasing further in subjects receiving mucolytics for 6-months. Data concerning the effect of GCS and antibiotics on the levels of ***Leukotriene B4*** (LTB4) are controversial [29,34,36].

Despite the abovementioned problems of traceability, pulmonary sampled ***cytokines*** have been extensively studied in the context of ECOPD. Of interest is the rather distinct pattern of TNF-α whose levels at day 5 are lower than those at day 14 [32]. This evolution pattern has also been described for systemic TNF-α and could provide evidence of a sustained inflammatory reaction during the late recovery period [37]. Concerning clinical variables, IL-8 at ECOPD onset correlated inversely with PFTs [25] whereas in regard to treatment interventions, clinical interpretation of published data should be done with caution in the absence of double-blind placebo-controlled trials. Small statistically significant differences in the concentrations of IL-6 have been observed after a 2 week antibiotic treatment and were followed by a further reduction after a 6-month course of mucolytics [31]. Interestingly, TNF-α levels at ECOPD onset did not differ according to the use of ICS [24], but at day 60 post hospitalization lower TNF-α levels have been found in patients on ICS therapy [32].

### Spontaneous sputum

Most of the SS biomarkers studied during ECOPD (Additional file 1: Table S1 and S2) were evaluated for their associations with the clinical severity and the causal diagnosis. Neutrophil elastase (NE) and some antimicrobial peptides correlated with clinical severity scores [38-40], whereas the magnitude of IL-8 rise from baseline correlated with symptom recovery time [41]. Persistent symptoms were paralleled by persistently elevated levels of IL-8, TNF-α and NE, and clinical resolution was related to their decrease to pre-ECOPD levels [39].

Sputum purulence has long been used for therapeutic decisions [42] but its association with the microbiologic yield has been controversial [43-45]. Published studies report that in ECOPD sputum myeloperoxidase (MPO) and IL-8 are associated with sputum color and purulence [44]. Moreover, MPO has been associated with the sputum leucocyte count [46], IL-8 with sputum bacterial load and the polymorphonuclear macrophages count [46], whereas the log of LTB4 concentration has been associated with the overall chemotactic activity of sputum [47].

As far as specific pathogens are concerned, published data are of particular interest. In a cohort of 50 patients studied during 6 years, acquisition of *H. influenza* and *M.catarrhalis* was associated with changes in SS levels of antimicrobial peptides and distinct trends of change were observed at ECOPD as compared to colonization [40]. The study of Dal Negro et al. used a two stage logistic model to identify the cause of an ECOPD. At the first decisional step, TNF-α enabled the recognition of ECOPD associated with the presence of *Ps.aeruginosa*, whereas at the second decisional step IL-8 and IL-1β discriminated patients with bacterial causes from those with a viral cause and from the non-infected ones [48]. Another study, reported that, sputum NE levels of 350 mU/ml had a sensitivity of 70.6%, a specificity of 84.2%, a positive predictive value of 88.9% and a negative predictive value of 61.5% in distinguishing *H. influenza* or *M.catarrhalis* from non bacterial ECOPD [38]. In a prospective study of the same group, TNF-α had the largest area under the curve for the identification of new strain ECOPD whereas combinations of sputum TNF-α, sputum NE and serum CRP performed better than any single biomarker [39]. Finally, following treatment, the decrease of IL-8, MPO, LTB4 and albumin leakage was more substantial after bacterial eradication [49].

## Semi-invasive sampling

### Induced sputum

Sputum induction is used in clinical practice for microbiological and cell count studies, whereas measurement of inflammatory biomarkers is increasingly implemented in research. Methodological issues that may influence the measurement of biomarkers, such as sample pre-treatment with dithiothreitol, have been an issue of discussion in the literature [50]. Two studies that evaluated the safety profile of sputum induction during ECOPD support the safety of its proper use in this setting [51,52].

Several biomarkers have been measured in the supernatants of induced sputum (Additional file 1: Table S1 and S2) but most of the groups studied ***cytokine*** levels, notably IL-6, IL-8 and TNFα. As compared to stable COPD or to baseline, IL-6 [53,54] and TNFa [55] levels were increased at ECOPD onset but this was not invariably observed for IL-8 [53-55]. During ECOPD, high levels of IL-6 have been associated with the presence of common cold symptoms [53] and a higher change from baseline in IL-6 concentration has been associated with a rhinovirus infection [54]. These findings were supported by a study evaluating an experimental virus infection of COPD patients, in which peak viral load correlated positively with peak sputum IL-6, IL-8, NE and TNF-α as well as with peak serum CRP levels [56]. On the other hand, although data are partly controversial [55,57], bacterial exacerbations were associated with increased IL-6, IL-8, TNFα, LTB4 and MPO levels [57], and TNFα was the best predictor of a bacterial ECOPD when compared to CRP and induced sputum MPO [57].

During recovery, some studies described a reduction of the levels of IL-6, IL-8 and TNFα under bronchodilators and systemic GCS with or without antibiotics [15,55], whereas other studies did not demonstrate a reduction of IL-6 and TNFα levels even 3 months after hospital discharge [16]. The adjunction of theophylline was associated with further reductions in IL-8 and TNFα, suggesting that theophylline may potentially enhance the anti-inflammatory action of standard treatment, notably that of systemic GCS [15]. Finally, budesonide and formoterol resulted in a significantly larger decrease in mRNA expression of IL-5 as compared to placebo [58].

### Spontaneous and induced sputum data analyzed in conjunction

As illustrated before, sputum analysis has been an area of intensive research. Sputum purulence and sputum cell counts have been particularly promising and were studied for their potential to characterize biological phenotypes of ECOPD and predict response to treatment [59,60]. Differences in the etiology of ECOPD but also in the severity of COPD or in treatment may modify the inflammatory response during ECOPD and notably the presence or not of sputum eosinophilia [53,61]. Concerning the analysis of biological molecules, several studies analyzed in conjunction data obtained from spontaneous and induced sputum (Additional file 1: Table S1 and S2). Although, in ECOPD, both sampling methods resulted in comparable microscopic purulence, salivary contamination and rate of isolation of major pathogens [62], studies comparing biomarkers between the two methods are scarce [38]. As different methodologies may be considered a confounding factor, these data are discussed separately in this section.

At ECOPD onset, the change in sputum IL-8 and IL-6 from baseline is inversely related to the baseline FEV_1_ indicating that patients with more severe COPD exhibit greater rises in inflammation at ECOPD [63]. Moreover, secretory leukocyte protease inhibitor (SLPI) correlates inversely with sputum polymorphonuclear leukocytes, and within the first 48 h of treatment a fall of the sputum polymorphonuclear count by 70% is accompanied by an increase in sputum SLPI and reductions of IL-8 and TNF-α [64].

In regard to the etiology of ECOPD, increased sputum IL-8 and TNF-α was associated to bacterial infection [64], whereas the rise of sputum IL-8 from baseline correlated with the rise in the airway bacterial load [63]. In the study of Bafadhel and coworkers a panel of biomarkers was evaluated in regard to four ECOPD phenotypes (bacteria-predominant, virus-predominant, sputum eosinophil-predominant and pauci-inflammatory exacerbations). These phenotypes could not be distinguished according to Anthonisen’s criteria and no single biomarker had an area under the curve greater than 0.70 in diagnosing an ECOPD. However, sputum IL-1b and serum CRP performed better in determining bacteria-associated ECOPD. For sputum IL-1b a cutoff point of 125 pg/ml had a sensitivity of 90% and a specificity of 80%, performing better than CRP [59].

## Invasive sampling

### Bronchoalveolar lavage

BAL obtained through bronchoscopy permits the study of cellular and biochemical components present in the epithelial lining fluid. So far only a few studies assessed simultaneously BAL and other airway sampling techniques in ECOPD [24,65,66]. Based on current evidence, the clinical usefulness of BAL biomarkers in ECOPD is rather limited. BAL biomarkers may illustrate the underlying mechanisms of ECOPD [24,65,67-70], but methodological issues such as low sample numbers or sample manipulation techniques have been considered to be confounding factors limiting the statistical significance of the results [24,65].

### Biomarkers of bronchial biopsies obtained during ECOPD

Biomarkers expressed in biopsies obtained during ECOPD (Additional file 1: Table S3) have been evaluated principally for their potential to act as neutrophil [71] or eosinophil [72-74] chemoattractants but associations with clinical variables or outcomes have not been sufficiently assessed. In this regard, in patients suffering from severe ECOPD, increased neutrophilia has been associated with an upregulation of the gene expression of two cytokines (epithelial-derived neutrophil attractant 78 and IL-8) and the expression of receptors CXCR1 and CXCR2. Although no association has been found between the number of neutrophils and the presence of a viral infection, subjects with evidence of viral infection had fewer CXCR1 m-RNA positive cells [71].

## Insights from stable COPD sampling

Pulmonary biomarkers sampled during stable COPD have been assessed for their potential to predict an imminent ECOPD and to identify patients at risk for frequent ECOPD (Table 3). Some molecules, such as FeNO [75], exhaled volatile compounds [76], sputum MPO [77] and BAL IL-8 [78], are promising but their clinical usefulness is not yet established due to the lack of large studies. BAL neutrophilia has been associated with an increased frequency of ECOPD [79], whereas sputum eosinophilia appears to be more promising than specific biomarkers in identifying patients at risk for an ECOPD [76,77], those that might benefit from the introduction of ICS [80] and those who could actually stop ICS without increasing the risk for an early exacerbation [77].

**Table 3:** Lung biomarkers measured during clinically stability (baseline) as predictors of ECOPD frequency

**Sample**	**Biomarker**	**Ref.**	**Comment**
**Exhaled air**	**FeNO**	[81]	↔: frequent (≥3/year), infrequent (≤2/year) ECOPD
		[75]	Intra-individual FeNO variability is positively associated with the ECOPD frequency
			eNOCoV ≥ 40%: twofold increase in ECOPD rate as compared to COPD with eNOCoV <40%*
	**VOCs**	[76]	Several compounds were associate with the number of ECOPD in the previous year
**EBC**	**pH**	[82]	No significant correlation with ECOPD frequency over the following 6M
**Spontaneous sputum**	**Elastase**	[83]	↔: frequent (≥3/year), infrequent (≤2/year) ECOPD
	**IL-8**	[83]	↔: frequent (≥3/year), infrequent (≤2/year) ECOPD
	**LTB4**	[83]	↔: frequent (≥3/year), infrequent (≤2/year) ECOPD
	**MPO**	[83]	↔: frequent (≥3/year), infrequent (≤2/year) ECOPD
	**Protein leakage**	[83]	↔: frequent (≥3/year), infrequent (≤2/year) ECOPD
**Induced sputum**	**ECP**	[77]	Not statistically significant hazard for ECOPD after cessation of ICS
	**IL-6**	[53]	Correlated with the frequency of ECOPD
	**IL-8**	[84]	Correlated with the total bacterial count. Bacterial colonization at baseline was associated with ECOPD frequency
		[53]	Correlated with the frequency of ECOPD
	**LTB4**	[77]	Not statistically significant hazard for ECOPD after cessation of ICS
	**MPO**	[77]	In the monovariate analysis (but not in the multivariate analysis) sputum MPO per neutrophil was a significant hazard for ECOPD after cessation of ICS. MPO level per se were not a significant hazard.
	**SLPI**	[83]	Negative correlation with ECOPD frequency over the preceding year
		[84]	Lower levels in samples colonized with a possible pathogen. Bacterial colonization in the stable state was associated with increased frequency of ECOPD.
**Induced and spontaneous sputum**	**ET-1**	[85]	ET-1 at stability and the rise of ET-1 during ECOPD did not correlate with the frequency of ECOPD
	**IL-6**	[86]	Patients with frequent ECOPD (≥2.52/y) had a faster rise over time in sputum IL6
	**IL-8**	[86]	No significant relation to exacerbation frequency
**Small volume lavage of the large airways**	**Albumin**	[87]	↔: patients with ≥3 antibiotic treated ECOPD during the past 2 years, patients without recurrent ECOPD
	**ECP**	[87]	↔: patients with ≥3 antibiotic treated ECOPD during the past 2 years, patients without recurrent ECOPD
	**Hyaluronan**	[87]	Not statistically significant difference in regard to recurrent ECOPD
	**IL-6**	[87]	↔: patients with ≥3 antibiotic treated ECOPD during the past 2 years, patients without recurrent ECOPD
	**IL-8**	[87]	↔: patients with ≥3 antibiotic treated ECOPD during the past 2 years, patients without recurrent ECOPD
	**MPO**	[87]	↔: patients with ≥3 antibiotic treated ECOPD during the past 2 years, patients without recurrent ECOPD
	**Tryptase**	[87]	↔: patients with ≥3 antibiotic treated ECOPD during the past 2 years, patients without recurrent ECOPD
**BAL**	**MPO**	[78]	↔: frequent (≥3/year), infrequent (<3/year) ECOPD
	**IL-8**	[78]	Higher levels in patients with frequent ECOPD, 1 pg/ml increase in IL-8 was associated with 1fold increase in the risk of frequent ECOPD
	**NE**	[78]	↔: frequent (≥3/year), infrequent (<3/year) ECOPD
	**TNF-α**	[78]	↔: frequent (≥3/year), infrequent (<3/year) ECOPD

Data concerning mRNA expression of biomarkers were not included in this table.

*Abbreviations:eNOCoV* intra-individual FeNO coefficient of variation, *ECP* eosinophil cationic protein, *ET-1* endothelin 1, *FeNO* exhaled nitric oxide, *ICS* inhaled corticosteroids, *IL* interleukin, *LTB4* leukotriene B4, *MPO* myeloperoxidase, *NE* neutrophil elastase, *SLPI* secretory leukoprotease inhibitor, *TNFα* tumor necrosis factor alpha, VOCs: volatile organic compounds.

**Symbols:** ↔: no difference.

*The FeNO monthly intra-subject variability was retrospectively assessed by calculating the CoV (mean/SD)x100.

## Promising biomarkers and future challenges

As in the case of systemic biomarkers in ECOPD [7], several pulmonary biomarkers are promising but none can be considered validated enough to gain its way into everyday clinical practice. So far no single biomarker can reliably identify an ECOPD at its onset, differentiate different gravities of ECOPD or consistently predict an unfavorable outcome, but most of the studied biomarkers have the potential to illustrate the clinical course of disease through the modification of their levels and to provide information on the underlying mechanisms of disease. Some molecules correlate with clinical variables, whereas a few may predict outcomes, direct toward a causal diagnosis or provide information concerning the response to treatment (Table 4). Pinpointing the most promising biomarkers and highlighting some clinically relevant conclusions, we may mention the following:

■ Although based on a single study, FeNO on admission may predict a significant post-treatment increase in FEV_1_. An optimum cut-off point of 26.8 ppb had a sensitivity of 74% and a specificity of 75% [12].

■ Among sputum biomarkers, the following ones appear more promising:

o Spontaneous sputum NE, IL-8 and TNF-a may reflect clinical severity and symptomatic recovery [38,39,41].

o Spontaneous sputum antimicrobial peptides (lysozyme, LL37 and SLPI) were associated to the acquisition of *H. influenza* and *M. catarrhalis,* and distinct trends of change were observed at ECOPD as compared to colonization [40].

o Spontaneous sputum TNF-α, IL-8, NE and IL-1β as well as induced sputum IL-6, IL-8, TNFα, LTB4 and MPO directed towards an etiological diagnosis [38,48,57,59,64]. Among induced sputum biomarkers TNFα was the best predictor of a bacterial ECOPD [57], whereas sputum IL-1b performed better than CRP in determining bacteria-associated ECOPD [59].

o The decrease of spontaneous sputum IL-8, MPO, LTB4 and albumin leakage was more substantial when bacterial eradication was achieved [49].

■ IL-6 was associated with a rhinovirus infection both in induced sputum [54,56] and in BAL [67].

■ Combinations of different pulmonary and/or systemic biomarkers may perform better than single biomarkers in identifying the causal etiology of ECOPD [39,59].

■ Current evidence on the clinical usefulness of exhaled breath condensate and BAL biomarkers in ECOPD is limited.

■ Several biomarkers sampled during stable COPD have the potential to identify patients at risk for frequent ECOPD, whereas patients with more severe COPD may exhibit greater rises in inflammation at ECOPD [63].

**Table 4:** Studies assessing biomarkers for their potential to provide clinically relevant information

	**Assessed biomarkers**
**Clinical variables**	
Symptoms at ECOPD onset	FeNO[14], EBC: H_2_O_2_[28], IS: IL-6[53], Nasal wash: IL-6[46], IL-8[46]
Clinical severity	EBC: CysLTs[29], H_2_O_2_[29], 8-isoprostane[29], LTB_4_[29], PGE_2_[29]
	SS: lactoferrin[40], LL-37[40], lysozyme[40], NE[31,38], SLPI[40]
	Large airway secretions: IL-8[65], TNF-α[65]
PFTs	FeNO[10,12,13,20]
	EBC: CysLTs[29], H_2_O_2_[29], 8-isoprostane[29], IL-8[25], LTB_4_[29], MPO[23], PGE_2_[29], pH[13], SLPI[23]
	IS/SS: IL-6[63], IL-8[63]
ABG analysis	FeNO[13]
	EBC: pH[13]
**Prediction of clinical outcomes**	
Symptomatic recovery	SS: IL-8[39,41], NE[39], TNF-α[39]
Length of hospital stay	FeNO[12]
**Causal diagnosis**	FeNO[18]
	SS: IL-1β[48], IL-8[38,46,48], LL37[40], lysozyme[40], NE[38,39], SLPI[40], TNFα[38,39,48]
	IS: IL-6[54,56,57], IL-8[55-57], LTB4[57], MPO[55,57], NE[56], TNF-α[55-57]
	IS/SS: IL-1β[59], IL-8[63,64], TNF-α[64]
	NW: IL-6[46]
	BAL: IL-6[68], IL-8[68], NE[68]
**Response to treatment**	FeNO[10,15]
	EBC: Cys-LTs[29], H_2_O_2_[29], IL-6[31], 8-isoprostane[29,31], LTB_4_[29], PGE_2_[29], TNFα[24,36]
	IS: IL-8[15], TNFα[15,34,36]

*Abbreviations*: *ABG* arterial blood gas, *BAL* bronchoalveolar lavage, *Cys-LTs* cysteinyl-leukotrienes, *EBC* exhaled breath condensate, FeNO: fractional exhaled nitric oxide, *H*_*2*_*O*_*2*_ hydrogen peroxide, *IL* interleukin, *IS/SS* induced and spontaneous sputum analyzed in conjunction, *LTB4* leukotriene B4, *MPO* myeloperoxidase, *NE* neutrophil elastase, *PFTs* pulmonary function tests, *PGE*_*2*_ prostaglandin E_2_, *SLPI* secretory leukoprotease inhibitor, *SS* spontaneous sputum, *TNFα* tumor necrosis factor alpha.

As already illustrated, biomarker discovery has become an area of intense investigation during the recent years. Collaborative efforts to standardize the procedure of biomarker development have identified 6 conceptual steps (discovery, qualification, verification, research assay optimization, biomarker validation and commercialization) [3,88,89]. In this context, the STARD statement and its proposed flowchart are useful resources that may improve the diagnostic accuracy of studies [90]. As a general framework, the aforementioned steps should also be implemented in the study of ECOPD biomarkers, especially when innovating sampling techniques are used. Currently, the majority of pulmonary biomarkers studied during ECOPD are at the initial steps of discovery and qualification, whereas only a limited number of studies have proceeded towards the subsequent steps of biomarker validation. The most advanced evaluation, so far, concerns the use of sputum biomarkers for the etiological diagnosis of ECOPD but the methodological heterogeneity of the conducted studies renders generalized data interpretation challenging. In order to obtain clinically applicable biomarkers, future studies should go beyond the simplistic description of biomarker kinetics and provide clinically oriented data. In parallel to the conduction of meticulous methodological studies, clinical decision studies are needed. Modern sequencing techniques such as metabolomics, proteomics or genomics are particularly promising and may help identify phenotype specific biomarkers. However, in complex diseases where redundant, synergistic or antagonistic mechanisms exist it becomes clear that the identification of clinically relevant biomarkers will depend on our ability to integrate data from disparate sources such as patient medical records and demographics. In this context, data mining strategies and computational models will be increasingly used for the extraction of clinically relevant information.

## Conclusions

In the current review, we summarized the published evidence regarding pulmonary biomarkers studied in the context of ECOPD and attempted to highlight their clinical relevance. Several biomarkers hold promise for improving the understanding of the complex mechanisms propagating ECOPD, several molecules correlate with clinical variables and a few are associated with clinical outcomes. Most of the evidence on the effect of treatment is based on a limited number of open-label, single-center studies that are characterized by a small sample size and an undefined statistical power. Despite these promising results, on the basis of the published evidence, no single molecule can be proposed yet for wide use in clinical practice. Further experimental studies and large scale clinical trials that incorporate biomarkers in decisional algorithms are required.

## Abbreviations

ABG: Arterial blood gas; BAL: Bronchoalveolar lavage; COPD: Chronic obstructive pulmonary disease; EBC: Exhaled breath condensate; ECOPD: Exacerbations of chronic obstructive pulmonary disease; FeNO: Fractional exhaled nitric oxide; GCS: Glucocorticosteroids; GSH: Glutathione; H2O2: Hydrogen peroxide; ICS: Inhaled corticosteroids; IL: Interleukin; IS: Induced sputum; LTB4: Leukotriene 4; MPO: Myeloperoxidase; NE: Neutrophil elastase; PFTs: Pulmonary function tests; SLPI: Secretory leukocyte protease inhibitor; SS: Spontaneous sputum.

## Competing interests

None of the authors has any conflict of interest related to the present manuscript.

## Authors’ contributions

AK conceived of the review and drafted the manuscript. KK revised the manuscript for important intellectual content. LPN revised the manuscript for important intellectual content. JWF revised the manuscript for important intellectual content. All authors read and approved the final manuscript.

## Supplementary Material

Additional file 1 Online supplement. Click here for file
