# Supplementary material for: Pulmonary biomarkers in COPD exacerbations: a systematic review
Source: Respir Res. 2013 Oct 21;14(1):111. doi: 10.1186/1465-9921-14-111 (PMC4014989; doi:10.1186/1465-9921-14-111)
Supplement: Additional file 1 — Online supplement. [file 1465-9921-14-111-S1.docx]

**ONLINE SUPPLEMENT**

**Pulmonary Biomarkers in COPD Exacerbations: a Systematic Review**

Angela Koutsokera, MD^1^, [angela.koutsokera@chuv.ch](mailto:angela.koutsokera@chuv.ch)

Konstantinos Kostikas, MD^2^, [ktk@otenet.gr](mailto:ktk@otenet.gr)

Laurent P. Nicod, MD^1^, [laurent.nicod@chuv.ch](mailto:laurent.nicod@chuv.ch)

Jean-William Fitting, MD^1^ jean-william.fitting@chuv.ch

From the ^1^Department of Respiratory Medicine, University Hospital of Lausanne, Switzerland; ^2^2^nd^ Respiratory Medicine Department, University of Athens Medical School, Attikon Hospital, Athens, Greece

**Correspondence to:**

Angela Koutsokera

Service de Pneumologie, Centre Hospitalier Universitaire Vaudois

Rue du Bugnon 46, 1011 Lausanne, Switzerland

Email: angela.koutsokera@chuv.ch

**Table S1: Cross-sectional studies assessing sputum biomarkers at ECOPD.**

| **Biomarker** | **Ref.** | **Sample** | **Comparisons at ECOPD onset** |
| --- | --- | --- | --- |
| ΑAT | ^[^[^1^](#_ENREF_1)^]^ | S | ECOPD without AAT deficiency>ECOPD with deficiency |
| IL-1β | [[2](#_ENREF_2)] | I | ECOPD>controls (smokers, non-smokers) |
| IL-6 | [[3](#_ENREF_3)] | S | ↔: ECOPD, stable COPD |
|  | [[4](#_ENREF_4)] | I | ECOPD>stable^∞^ |
|  | [[5](#_ENREF_5)] | I | ↔: ECOPD, controls (current, ex-smokers) |
|  | [[2](#_ENREF_2)] | I | ECOPD>controls (smokers, non-smokers) |
| IL-8 | [[6](#_ENREF_6)] | S | ECOPD with mucoid sputum<purulent sputum |
|  | [[7](#_ENREF_7)] | S | ECOPD>stable COPD |
|  | [[1](#_ENREF_1)] | S | ECOPD without deficiency>ECOPD with deficiency |
|  | [[8](#_ENREF_8)] | S | ECOPD> controls, stable COPD |
|  | [[4](#_ENREF_4)] | I | ↔: ECOPD, stable^∞^ |
|  | [[2](#_ENREF_2)] | I | ECOPD>controls (smokers, non-smokers) |
|  | [[9](#_ENREF_9)] | I | ECOPD> non-smokers, asymptomatic smokers, symptomatic smokers |
|  | [[10](#_ENREF_10)] | I | Median level 690pg/ml in ECOPD, undetectable in most non-smokers and smokers |
|  | [[5](#_ENREF_5)] | I | ↔: ECOPD, controls (current, ex-smokers) |
| IL-10 | [[2](#_ENREF_2)] | I | ECOPD>controls (smokers, non-smokers) |
| 8-isoprostane | [[10](#_ENREF_10)] | I | ECOPD< non-smokers, asymptomatic smokers, symptomatic smokers |
| LTB4 | [[6](#_ENREF_6)] | S | ECOPD with mucoid sputum <purulent sputum |
|  | [[7](#_ENREF_7)] | S | ECOPD>stable COPD |
|  | [[1](#_ENREF_1)] | S | ECOPD without AAT deficiency<ECOPD with deficiency |
| MMP-9 | [[11](#_ENREF_11)] | S | ECOPD> controls, smokers, stable COPD |
|  | [[12](#_ENREF_12)] | N/R | ECOPD>controls (the exact time point of sampling N/R) |
| MMP-8 | [[13](#_ENREF_13)] | I | ECOPD> controls, smokers, stable COPD |
| MPO | [[1](#_ENREF_1)] | S | ↔: ECOPD without AAT deficiency, ECOPD with deficiency |
|  | [[3](#_ENREF_3)] | S | ECOPD>stable^∞^ |
|  | [[7](#_ENREF_7)] | S | ECOPD>stable |
|  | [[6](#_ENREF_6)] | S | ECOPD with mucoid sputum <purulent sputum |
| NE | [[6](#_ENREF_6)] | S | ECOPD with mucoid sputum <purulent sputum |
|  | [[1](#_ENREF_1)] | S | ECOPD without AAT deficiency<ECOPD with deficiency |
|  | [[13](#_ENREF_13)] | I | ECOPD> controls, smokers, stable COPD |
|  | [[5](#_ENREF_5)] | I | ↔: ECOPD, controls (current, ex-smokers), but ECOPD on D9, 12,15>controls |
| SLPI | [[1](#_ENREF_1)] | S | ECOPD without AAT deficiency>ECOPD with deficiency |
|  | [[8](#_ENREF_8)] | S | ECOPD<controls, stable COPD |
|  | [[2](#_ENREF_2)] | I | ECOPD>controls (smokers, non-smokers) |
| TNFα | [[8](#_ENREF_8)] | I+S | ECOPD>controls, stable COPD |
|  | [[14](#_ENREF_14)] | I | ECOPD>controls (smokers, non-smokers) |
|  | [[2](#_ENREF_2)] | I | ECOPD>controls (smokers, non smokers) |
|  | [[5](#_ENREF_5)] | I | ↔: ECOPD, controls (current, ex-smokers) |
| Stnf-R55 | [[14](#_ENREF_14)] | I | ECOPD>controls (smokers, non smokers) |
| Stnf-R75 | [[14](#_ENREF_14)] | I | ECOPD>controls (smokers, non smokers) |

**Abbreviations:** AΑΤ: alpha 1 antitrypsin, D=day, I: induced sputum, I+S: some patients provided induced and some spontaneous sputum**,** IL: interleukin, LTB4: leukotriene B4, MMP: matrix metalloproteinase, MPO : myeloperoxidase, NE : neutrophil elastase, NTHI: non-typeable Haemophilus influenza, N/R: not reported, S: spontaneous sputum, SLPI : secretory leukocyte protease inhibitor, Stnf-R: soluble tumor necrosis factor receptor, TNFα: tumor necrosis factor alpha. **Symbols:** ↔: no difference, **∞**: some of the samples were paired

**Table S2: Longitudinal studies assessing sputum biomarkers at ECOPD.**

| **Biomarker** | **Ref.** | **Sample** | **Course:** | **Course:** | **Comment** |
| --- | --- | --- | --- | --- | --- |
|  |  |  | **From baseline to ECOPD onset** | **After ECOPD onset** |  |
| AΑΤ | [[1](#_ENREF_1)] | S | ↑ | ↓ | No GCS. ↓ by D3 in AAT deficient patients, by D28 in non deficient patients |
| CCL13 | [[15](#_ENREF_15)] | I+S | ↑ |  | 148 ECOPD events from 75 patients |
| IL-1β | [[15](#_ENREF_15)] | I+S | ↑ |  | 148 ECOPD events from 75 patients |
|  | [[2](#_ENREF_2)] | I |  | ↓ | GCS. ↓ by M3 after discharge |
| IL-4 | [[16](#_ENREF_16)] | I |  | ↓ | GCS for some. ↓ by W8 |
| IL-6 | [[17](#_ENREF_17)] | S | ↑ | ↓ | GCS for 67%. ↓ to baseline levels by D14 |
|  | [[18](#_ENREF_18)] | S |  | ↔ |  |
|  | [[9](#_ENREF_9)] | I |  | ↓ | GCS. ↓ by M3 |
|  | [[2](#_ENREF_2)] | I |  | ↔ | GCS. Did not reduce by M3 after discharge |
|  | [[19](#_ENREF_19)] | I | ↔ |  |  |
|  | [[4](#_ENREF_4)] | I | ↑^∞^ |  |  |
|  | [[20](#_ENREF_20)] | I | ↑ |  |  |
|  | [[5](#_ENREF_5)] | I | ↔: Baseline,D5 | ↔ | Experimental rhinovirus infection at D0. Assessment time points: Baseline, D5,9,12,15,21,28,35,42 |
|  | [[21](#_ENREF_21)] | I+S | ↔* | ↔* | GCS some |
|  | [[15](#_ENREF_15)] | I+S | ↑ |  | 148 ECOPD events from 75 patients |
| IFN-γ | [[16](#_ENREF_16)] | I |  | ↓ | GCS for some. ↓ by W8 |
| IL-5 | [[15](#_ENREF_15)] | I+S | ↔ |  | 148 ECOPD events from 75 patients |
| IL-6 | [[15](#_ENREF_15)] | I+S | ↑ |  | 148 ECOPD events from 75 patients |
| IL-6R | [[15](#_ENREF_15)] | I+S | ↑ |  | 148 ECOPD events from 75 patients |
| IL-8 | [[6](#_ENREF_6)] | S |  | ↔ | Mucoid sputum ECOPD: No change |
|  |  |  |  | Initial↓, ↑ | Purulent sputum ECOPD: ↓ by D5, ↑ afterwards |
|  | [[7](#_ENREF_7)] | S |  | ↓ | ↓ by D3 |
|  | [[1](#_ENREF_1)] | S | ↑ | ↓ | No GCS. ↓ by D3 (for patients with AAT deficiency) |
|  | [[3](#_ENREF_3)] | S |  | ↔^∞^ |  |
|  | [[17](#_ENREF_17)] | S | ↔ | ↓ | GCS for 67%. ↓ to baseline levels by D14 |
|  | [[22](#_ENREF_22)] | S | ↑ | ↓ |  |
|  | [[23](#_ENREF_23)] | S |  | Initial↓, ↑  ↔ | GCS for some. Group of bacterial eradication: ↓ by D10 but at 2M levels were similar with levels at D1  Group of bacterial persistence: no change |
|  | [[20](#_ENREF_20)] | I | ↑ |  |  |
|  | [[24](#_ENREF_24)] | I | ↑ | ↓ | GCS for some. ↓ by M1 |
|  | [[2](#_ENREF_2)] | I |  | ↓ | GCS. ↓ by M3 after discharge |
|  | [[9](#_ENREF_9)] | I |  | ↓ | GCS. ↓ by M3 |
|  | [[25](#_ENREF_25)] | I |  | ↓ | GCS. ↓ by 16 weeks after remission |
|  | [[19](#_ENREF_19)] | I | ↔ |  |  |
|  | [[4](#_ENREF_4)] | I | ↔^∞^ |  |  |
|  | [[5](#_ENREF_5)] | I | ↔: Baseline,D5 | ↑over baseline levels on D9 | Increased on D9. Experimental rhinovirus infection at D0. Assessment timepoints: Baseline, D5,9,12,15,21,28,35,42 |
|  | [[18](#_ENREF_18)] | I+S | ↑ |  |  |
|  | [[8](#_ENREF_8)] | I+S |  | ↓ | GCS. ↓ by D2 |
|  | [[21](#_ENREF_21)] | I+S | ↔* | ↔* | GCS for some |
|  | [[26](#_ENREF_26)] | I+S | ↑ |  |  |
|  | [[15](#_ENREF_15)] | I+S | ↔ |  | 148 ECOPD events from 75 patients |
|  | [[27](#_ENREF_27)] | S |  |  | Levels 8w post ECOPD, where higher in those with purulent sputum ECOPD |
| IL-10 | [[2](#_ENREF_2)] | I |  | ↓ | GCS. ↓ by M3 after discharge |
| IP-9 (CXCL11) | [[15](#_ENREF_15)] | I+S | ↔ |  | 148 ECOPD events from 75 patients |
| IP-10 (CXCL10) | [[15](#_ENREF_15)] | I+S | ↔ |  | 148 ECOPD events from 75 patients |
| ECP | [[28](#_ENREF_28)] | I |  | ↓ | GCS. ↓ by W8-10 |
|  | [[19](#_ENREF_19)] | I | ↑ |  |  |
|  | [[25](#_ENREF_25)] | I |  | ↔ | GCS |
|  | [[26](#_ENREF_26)] | I+S | ↑ |  |  |
| Eotaxin (CCL26) | [[26](#_ENREF_26)] | I+S | ↔ |  |  |
| ET-1 | [[21](#_ENREF_21)] | I+S | ↔* | ↔* | GCS for some |
| GM-CSF | [[25](#_ENREF_25)] | I |  | ↔ | GCS |
| Lactoferin | [[29](#_ENREF_29)] | S | ↔* | ↔* | 153 samples from 11 patients collected over 6 years. No difference between baseline (culture-negative samples), colonization and ECOPD with NTHI or M catarrhalis. |
| LL-37 | [[29](#_ENREF_29)] | S | ↑* | ↑* | 153 samples from 11 patients collected over 6 years. Increased levels at ECOPD due to NTHI and M catarrhalis as compared to baseline (culture negative samples). Higher levels at ECOPD as compared to colonization. |
| Lysozyme | [[29](#_ENREF_29)] | S | ↓* | ↓* | 153 samples from 11 patients collected over 6 years. Reduced levels at ECOPD due to NTHI or M catarrhalis as compared to baseline (culture negative samples). Similar levels at ECOPD and colonization. |
| LTB4 | [[6](#_ENREF_6)] | S |  | ↔ | ECOPD with mucoid sputum: No change |
|  |  |  |  | ↓ | ECOPD with purulent sputum: ↓ by D5 |
|  | [[7](#_ENREF_7)] | S |  | Initial↓,↑ | ↓ by D5, small rise thereafter |
|  | [[1](#_ENREF_1)] | S | ↑ | ↓ | GCS No. ↓ by D3 in AAT deficient patients, by D28 in non deficient patients |
|  | [[23](#_ENREF_23)] | S |  | ↓ | GCS some. Group of bacterial eradication: ↓ by D10 |
|  |  |  |  | ↔ | Group of bacterial persistence: no change |
|  | [[19](#_ENREF_19)] | I | ↑ |  |  |
|  | [[27](#_ENREF_27)] | S |  |  | Levels 8w post ECOPD: no difference between those with purulent and non-purulent sputum ECOPD |
| MCP-I (CCL2) | [[19](#_ENREF_19)] | I | ↑ |  |  |
|  | [[15](#_ENREF_15)] | I+S | ↔ |  | 148 ECOPD events from 75 patients |
| MIP-1a (CCL3) | [[15](#_ENREF_15)] | I+S | ↔ |  | 148 ECOPD events from 75 patients |
| MIP-1β (CCL4) | [[15](#_ENREF_15)] | I+S | ↑ |  | 148 ECOPD events from 75 patients |
| MMP-9 | [[11](#_ENREF_11)] | I | ↑ |  |  |
| MMP-8 | [[13](#_ENREF_13)] | I |  | ↓ | GCS. ↓ by W4 |
| MPO | [[1](#_ENREF_1)] | S | ↑ | ↓ | No GCS. ↓ by D3 in AAT deficient patients, by D28 in non deficient patients |
|  | [[3](#_ENREF_3)] | S |  | ↓^∞^ |  |
|  | [[7](#_ENREF_7)] | S |  | ↓ |  |
|  | [[23](#_ENREF_23)] | S |  | ↓ | GCS for some. Group of bacterial eradication: ↓ by D10 |
|  |  |  |  | ↔ | Group of bacterial persistence: no change |
|  | [[6](#_ENREF_6)] | S |  | Initial↓, ↑ | ECOPD with mucoid sputum: ↓ by D5, ↑ by D56 |
|  |  |  |  | ↓ | ECOPD with purulent sputum: ↓ by D5 |
|  | [[24](#_ENREF_24)] | I | ↔ | ↔ | GCS some |
|  | [[19](#_ENREF_19)] | I | ↔ |  |  |
|  | [[25](#_ENREF_25)] | I |  | ↓ | GCS Yes. ↓ by W16 after remission |
|  | [[27](#_ENREF_27)] | S |  |  | Levels 8w post ECOPD: no difference between those with purulent and non-purulent sputum ECOPD |
| NE | [[6](#_ENREF_6)] | S |  | ↔ | ECOPD with mucoid sputum: no change |
|  |  |  |  | ↓ | ECOPD with purulent sputum: ↓ by D5 |
|  | [[1](#_ENREF_1)] | S | ↑ | ↓ | GCS No. ↓ by D3 in AAT deficient patients, by D28 in non deficient patients |
|  | [[7](#_ENREF_7)] | S |  | ↓ | Undetectable by D5 |
|  | [[22](#_ENREF_22)] | S | ↑ | ↓ |  |
|  | [[23](#_ENREF_23)] | S |  | ↓ | GCS for some. ↓ by D10 |
|  | [[28](#_ENREF_28)] | I |  | ↓ | GCS. ↓ by W8-10 |
|  | [[13](#_ENREF_13)] | I |  | ↓ | GCS. ↓ by W4 |
|  | [[5](#_ENREF_5)] | I | ↔: Baseline,D5 | ↑over baseline levels on D9,15 | D9, 15 increased as compared to baseline. Experimental rhinovirus infection at D0. Assessment timepoints: Baseline, D5,9,12,15,21,28,35,42 |
|  | [[26](#_ENREF_26)] | I+S | ↑ |  |  |
|  | [[30](#_ENREF_30)] | N/R |  | ↓ | AAT deficient patients |
|  | [[31](#_ENREF_31)] | S |  | ↔ | No change by D56 |
| Neopterin | [[15](#_ENREF_15)] | I+S | ↔ |  | 148 ECOPD events from 75 patients |
| PR3 | [[31](#_ENREF_31)] | S |  | ↓ | From 12 samples, 10 had detectable activity at D1 and 6 at D56. |
| RANTES (CCL5) | [[26](#_ENREF_26)] | I+S | ↑ |  |  |
|  | [[15](#_ENREF_15)] | I+S | ↑ |  | 148 ECOPD events from 75 patients |
| SLPI | [[29](#_ENREF_29)] | S | ↓* | ↓* | 153 samples from 11 patients collected over 6 years. Reduced levels at ECOPD due to NTHI and M catarrhalis as compared to baseline (culture negative samples). Lower levels at ECOPD due to NTHI as compared to colonization. Similar levels at ECOPD due to M.catarrhalis as compared to colonization. |
|  | [[2](#_ENREF_2)] | I |  | ↔ | GCS. Did not reduce by M3 after discharge. |
| TAS | [[9](#_ENREF_9)] | I |  | ↓ | GCS Yes. ↓ by M3 |
| TARC (CCL17) | [[15](#_ENREF_15)] | I+S | ↑ |  | 148 ECOPD events from 75 patients |
| TIMP-1 | [[11](#_ENREF_11)] | N/R | ↔ |  |  |
| TNFα | [[22](#_ENREF_22)] | S | ↑ | ↓ |  |
|  | [[24](#_ENREF_24)] | I | ↑ | ↓ | GCS some. ↓ by M1 |
|  | [[9](#_ENREF_9)] | I |  | ↓ | GCS. ↓ by M3 |
|  | [[19](#_ENREF_19)] | I | ↔ |  |  |
|  | [[14](#_ENREF_14)] | I |  | ↓ | GCS avoided. ↓ post treatment |
|  | [[2](#_ENREF_2)] | I |  | ↔ | GCS. Did not reduce by M3 after discharge. |
|  | [[5](#_ENREF_5)] | I | ↔: Baseline,D5 | ↔ | Experimental rhinovirus infection at D0. Assessment timepoints: Baseline, D5,9,12,15,21,28,35,42 |
|  | [[8](#_ENREF_8)] | I+S |  | ↓ | GCS. ↓ by D2 |
|  | [[15](#_ENREF_15)] | I+S | ↑ |  | 148 ECOPD events from 75 patients |
| TNF-R1 | [[15](#_ENREF_15)] | I+S | ↑ |  | 148 ECOPD events from 75 patients |
| TNF-R2 | [[15](#_ENREF_15)] | I+S | ↑ |  | 148 ECOPD events from 75 patients |
| TNF-R55 | [[14](#_ENREF_14)] | I |  | ↓ | GCS avoided. ↓ post treatment |
| TNF-R75 | [[14](#_ENREF_14)] | I |  | ↓ | GCS avoided. ↓ post treatment |
| Tryptase | [[26](#_ENREF_26)] | I+S | ↔ |  |  |
| SLPI | [[23](#_ENREF_23)] | S |  | Initial↔,↑ | GCS some. Stable until D10, ↑ by 2M |
|  | [[1](#_ENREF_1)] | S | ↑ | ↑ | No GCS. ↑ by D3 in AAT deficient patients, by D28 in non deficient patients |
|  | [[8](#_ENREF_8)] | I+S |  | ↓ | GCS. Levels ↓ by D2 |

**Abbreviations:** ΑΑΤ: alpha 1-antitrypsin, MIP-1: macrophage inflammatory protein 1, I: induced sputum, I+S: some patients provided induced and some spontaneous sputum, IP: interferon-gamma inducible protein, D: day after ECOPD onset, ECP: eosinophil cationic protein, ET-1: endothelin 1, GCS: administration of systemic glucocorticosteroids, GM-CSF: granulocyte macrophage colony stimulating factor, IFN-γ: interferon gamma, IL: interleukin, LTB4: leukotriene B4, M: month after ECOPD onset, MCP: Monocyte chemoattractant protein, MMP: matrix metalloproteinase, MPO: myeloperoxidase, NE : neutrophil elastase, N/R: not reported, NTHI: nontypeable *Haemophilus influenza*, PR3: proteinase 3, RANTES: Regulated on activation normal T-cell expressed and secreted , SLPI: secretory leukocyte protease inhibitor, TARC: thymus and activation regulated chemokine, TNF-R: tumour necrosis factor receptor ,TAS: Total antioxidant status, TIMP-1: Tissue inhibitor of metalloproteinase, TNFα: tumor necrosis factor alpha, W: weeks after ECOPD onset.

**Symbols:** ↔: no difference, ↑: increase, ↓: decrease, S: spontaneous sputum, *: stability samples obtained before and after the ECOPD, **∞**: some of the samples were paired

**Table S3: Biomarkers of bronchial biopsies obtained from patients suffering from an ECOPD**

| **Biomarker** | **Ref.** | **Subjects** | **Comparisons** |
| --- | --- | --- | --- |
| AP-1 DNA binding activity | [[32](#_ENREF_32)] | 2 ECOPD, 2 COPD, 4 controls | ↔ |
| CCR3 expression | [[33](#_ENREF_33)] | 14 ECOPD, 20 asthma, 8 controls | ECOPD>controls, ↔: ECOPD, asthma |
| CysLT1 receptor expression | [[34](#_ENREF_34)] | 15ECOPD, 16 COPD, 15 controls | ECOPD:COPD, controls |
| ENA-78 (CXCL5) expression | [[35](#_ENREF_35)] | 15 ECOPD, 7 COPD, 15 controls | ECOPD>COPD, controls |
| E-selectin stained vessels | [[36](#_ENREF_36)] | 11 ECOPD, 12 COPD | ↔ |
| IL-8 (CXCL8) expression | [[35](#_ENREF_35)] | 15 ECOPD, 7 COPD, 15 controls | ECOPD>COPD, controls |
| IL-8 receptor α (CXCR1) expression | [[35](#_ENREF_35)] | 15 ECOPD, 7 COPD, 15 controls | ECOPD>COPD, controls |
| IL-8 receptor β (CXCR2) expression | [[35](#_ENREF_35)] | 15 ECOPD, 7 COPD, 15 controls | ECOPD>COPD, controls |
| EG2 expression (activated eosinophils) | [[36](#_ENREF_36)] | 11 ECOPD, 12 COPD | ECOPD>COPD |
|  | [[37](#_ENREF_37)] | 9 ECOPD, 11 COPD, 7 controls | ECOPD>COPD, controls |
|  | [[38](#_ENREF_38)] | 11 ECOPD,10 COPD, 18 asthma, 7 controls | ECOPD>COPD, controls, ↔: ECOPD, asthma |
| Eotaxin expression | [[37](#_ENREF_37)] | 9 ECOPD, 11 COPD, 7 controls | ECOPD>controls, ↔: ECOPD, COPD |
|  | [[33](#_ENREF_33)] | 14 ECOPD, 20 asthma, 8 controls | ECOPD>controls, ↔: ECOPD, asthma |
| ICAM-1 stained vessels | [[36](#_ENREF_36)] | 11 ECOPD, 12 COPD | ↔ |
| IL-1 beta positive cells | [[36](#_ENREF_36)] | 11 ECOPD, 12 COPD | ↔ |
| IL-2R positive cells | [[36](#_ENREF_36)] | 11 ECOPD, 12 COPD | ↔ |
| IL-4 expression | [[37](#_ENREF_37)] | 9 ECOPD, 11 COPD, 7 controls | ↔ |
| IL-5 expression | [[37](#_ENREF_37)] | 9 ECOPD, 11 COPD, 7 controls | ↔ |
|  | [[38](#_ENREF_38)] | 11 ECOPD,10 COPD, 18 asthma, 7 controls | Asthma>ECOPD, COPD, controls  ↔: ECOPD, COPD, Controls |
| MCP4 expression | [[37](#_ENREF_37)] | 9 ECOPD, 11 COPD, 7 controls | ↔ |
| Neutrophil elastase | [[35](#_ENREF_35)] | 15 ECOPD, 7 COPD, 15 controls | ECOPD>COPD, controls |
| NF-Κβ DNA binding activity | [[32](#_ENREF_32)] | 7 ECOPD, 3 COPD, 4 smokers, 7 controls | ↔ |
| RANTES (CCL5) | [[37](#_ENREF_37)] | 9 ECOPD, 11 COPD, 7 controls | ECOPD>COPD, controls |
| TNF-α positive cells | [[36](#_ENREF_36)] | 11 ECOPD, 12 COPD | ECOPD>COPD |
| VLA-1 positive cells | [[36](#_ENREF_36)] | 11 ECOPD, 12 COPD | ECOPD>COPD |

**Abbreviations:** ΑP-1: activator protein 1, CCR3: CC-chemokine receptor 3, EG-2: antieosinophil cationic protein, ENA-78: epithelial derived neutrophil attractant-78, IL: interleukin, MCP: monocyte chemoattractant protein, NF-κΒ: nuclear factor κΒ, RANTES: Regulated on activation normal T-cell expressed and secreted, TNF-α: tumour necrosis factor alpha, VLA-1: very late activation antigen-1

**Symbols:** ↔: no difference

**REFERENCES**

1. Hill AT, Campbell EJ, Bayley DL, Hill SL, Stockley RA: **Evidence for excessive bronchial inflammation during an acute exacerbation of chronic obstructive pulmonary disease in patients with alpha(1)-antitrypsin deficiency (PiZ).** *Am J Respir Crit Care Med* 1999, **160:**1968-1975.

2. Kersul AL, Iglesias A, Rios A, Noguera A, Forteza A, Serra E, Agusti A, Cosio BG: **Molecular mechanisms of inflammation during exacerbations of chronic obstructive pulmonary disease.** *Arch Bronconeumol* 2011, **47:**176-183.

3. Hurst JR, Perera WR, Wilkinson TM, Donaldson GC, Wedzicha JA: **Systemic and upper and lower airway inflammation at exacerbation of chronic obstructive pulmonary disease.** *Am J Respir Crit Care Med* 2006, **173:**71-78.

4. Bhowmik A, Seemungal TA, Sapsford RJ, Wedzicha JA: **Relation of sputum inflammatory markers to symptoms and lung function changes in COPD exacerbations.** *Thorax* 2000, **55:**114-120.

5. Mallia P, Message SD, Gielen V, Contoli M, Gray K, Kebadze T, Aniscenko J, Laza-Stanca V, Edwards MR, Slater L *et al*: **Experimental rhinovirus infection as a human model of chronic obstructive pulmonary disease exacerbation.** *Am J Respir Crit Care Med* 2011, **183:**734-742.

6. Gompertz S, O'Brien C, Bayley DL, Hill SL, Stockley RA: **Changes in bronchial inflammation during acute exacerbations of chronic bronchitis.** *Eur Respir J* 2001, **17:**1112-1119.

7. Crooks SW, Bayley DL, Hill SL, Stockley RA: **Bronchial inflammation in acute bacterial exacerbations of chronic bronchitis: the role of leukotriene B4.** *Eur Respir J* 2000, **15:**274-280.

8. Pant S, Walters EH, Griffiths A, Wood-Baker R, Johns DP, Reid DW: **Airway inflammation and anti-protease defences rapidly improve during treatment of an acute exacerbation of COPD.** *Respirology* 2009, **14:**495-503.

9. Cosio BG, Iglesias A, Rios A, Noguera A, Sala E, Ito K, Barnes PJ, Agusti A: **Low-dose theophylline enhances the anti-inflammatory effects of steroids during exacerbations of COPD.** *Thorax* 2009, **64:**424-429.

10. Mazur W, Stark H, Sovijarvi A, Myllarniemi M, Kinnula VL: **Comparison of 8-Isoprostane and Interleukin-8 in Induced Sputum and Exhaled Breath Condensate from Asymptomatic and Symptomatic Smokers.** *Respiration* 2009, **78:**209-216.

11. Mercer PF, Shute JK, Bhowmik A, Donaldson GC, Wedzicha JA, Warner JA: **MMP-9, TIMP-1 and inflammatory cells in sputum from COPD patients during exacerbation.** *Respir Res* 2005, **6:**151-160.

12. Huang JT, Chaudhuri R, Albarbarawi O, Barton A, Grierson C, Rauchhaus P, Weir CJ, Messow M, Stevens N, McSharry C *et al*: **Clinical validity of plasma and urinary desmosine as biomarkers for chronic obstructive pulmonary disease.** *Thorax* 2012, **67:**502-508.

13. Ilumets H, Rytila PH, Sovijarvi AR, Tervahartiala T, Myllarniemi M, Sorsa TA, Kinnula VL: **Transient elevation of neutrophil proteinases in induced sputum during COPD exacerbation.** *Scand J Clin Lab Invest* 2008,:1-6.

14. Zeng M, Wen Y, Liu LY, Wang H, Guan KP, Huang X: **Role of TNF-alpha, sTNF-R55 and sTNF-R75 in Inflammation of Acute Exacerbations of Chronic Obstructive Pulmonary Disease.** *Respiration* 2009, **78:**399-403.

15. Bafadhel M, McKenna S, Terry S, Mistry V, Reid C, Haldar P, McCormick M, Haldar K, Kebadze T, Duvoix A *et al*: **Acute exacerbations of chronic obstructive pulmonary disease: identification of biologic clusters and their biomarkers.** *Am J Respir Crit Care Med* 2011, **184:**662-671.

16. Makris D, Lazarou S, Alexandrakis M, Kourelis TV, Tzanakis N, Kyriakou D, Gourgoulianis KI: **Tc2 response at the onset of COPD exacerbations.** *Chest* 2008, **134:**483-488.

17. Perera WR, Hurst JR, Wilkinson TM, Sapsford RJ, Mullerova H, Donaldson GC, Wedzicha JA: **Inflammatory changes, recovery and recurrence at COPD exacerbation.** *Eur Respir J* 2007, **29:**527-534.

18. Wilkinson TM, Hurst JR, Perera WR, Wilks M, Donaldson GC, Wedzicha JA: **Effect of interactions between lower airway bacterial and rhinoviral infection in exacerbations of COPD.** *Chest* 2006, **129:**317-324.

19. Bathoorn E, Liesker JJ, Postma DS, Koeter GH, van der Toorn M, van der Heide S, Ross HA, van Oosterhout AJ, Kerstjens HA: **Change in inflammation in out-patient COPD patients from stable phase to a subsequent exacerbation.** *Int J Chron Obstruct Pulmon Dis* 2009, **4:**101-109.

20. Seemungal TA, Harper-Owen R, Bhowmik A, Jeffries DJ, Wedzicha JA: **Detection of rhinovirus in induced sputum at exacerbation of chronic obstructive pulmonary disease.** *Eur Respir J* 2000, **16:**677-683.

21. Roland M, Bhowmik A, Sapsford RJ, Seemungal TA, Jeffries DJ, Warner TD, Wedzicha JA: **Sputum and plasma endothelin-1 levels in exacerbations of chronic obstructive pulmonary disease.** *Thorax* 2001, **56:**30-35.

22. Sethi S, Wrona C, Eschberger K, Lobbins P, Cai X, Murphy TF: **Inflammatory profile of new bacterial strain exacerbations of chronic obstructive pulmonary disease.** *Am J Respir Crit Care Med* 2008, **177:**491-497.

23. White AJ, Gompertz S, Bayley DL, Hill SL, O'Brien C, Unsal I, Stockley RA: **Resolution of bronchial inflammation is related to bacterial eradication following treatment of exacerbations of chronic bronchitis.** *Thorax* 2003, **58:**680-685.

24. Aaron SD, Angel JB, Lunau M, Wright K, Fex C, Le Saux N, Dales RE: **Granulocyte inflammatory markers and airway infection during acute exacerbation of chronic obstructive pulmonary disease.** *Am J Respir Crit Care Med* 2001, **163:**349-355.

25. Tsoumakidou M, Tzanakis N, Chrysofakis G, Siafakas NM: **Nitrosative stress, heme oxygenase-1 expression and airway inflammation during severe exacerbations of COPD.** *Chest* 2005, **127:**1911-1918.

26. Fujimoto K, Yasuo M, Urushibata K, Hanaoka M, Koizumi T, Kubo K: **Airway inflammation during stable and acutely exacerbated chronic obstructive pulmonary disease.** *Eur Respir J* 2005, **25:**640-646.

27. Carter RI, Ungurs MJ, Mumford RA, Stockley RA: **Alpha-Val360: a marker of neutrophil elastase and COPD disease activity.** *Eur Respir J* 2013, **41:**31-38.

28. Papi A, Bellettato CM, Braccioni F, Romagnoli M, Casolari P, Caramori G, Fabbri LM, Johnston SL: **Infections and airway inflammation in chronic obstructive pulmonary disease severe exacerbations.** *Am J Respir Crit Care Med* 2006, **173:**1114-1121.

29. Parameswaran GI, Sethi S, Murphy TF: **Effects of bacterial infection on airway antimicrobial peptides and proteins in COPD.** *Chest* 2011, **140:**611-617.

30. Carter RI, Mumford RA, Treonze KM, Finke PE, Davies P, Si Q, Humes JL, Dirksen A, Piitulainen E, Ahmad A *et al*: **The fibrinogen cleavage product Aalpha-Val360, a specific marker of neutrophil elastase activity in vivo.** *Thorax* 2011, **66:**686-691.

31. Sinden NJ, Stockley RA: **Proteinase 3 activity in sputum from subjects with alpha-1-antitrypsin deficiency and COPD.** *Eur Respir J* 2013, **41:**1042-1050.

32. Drost EM, Skwarski KM, Sauleda J, Soler N, Roca J, Agusti A, MacNee W: **Oxidative stress and airway inflammation in severe exacerbations of COPD.** *Thorax* 2005, **60:**293-300.

33. Bocchino V, Bertorelli G, Bertrand CP, Ponath PD, Newman W, Franco C, Marruchella A, Merlini S, Del Donno M, Zhuo X *et al*: **Eotaxin and CCR3 are up-regulated in exacerbations of chronic bronchitis.** *Allergy* 2002, **57:**17-22.

34. Zhu J, Bandi V, Qiu S, Figueroa D, Evans JF, Barnes N, Guntupalli K, Jeffery PK: **CysLT1 receptor expression associated with bronchial inflammation in severe exacerbations of COPD.** *Chest* 2012, **142:**347-357

35. Qiu Y, Zhu J, Bandi V, Atmar RL, Hattotuwa K, Guntupalli KK, Jeffery PK: **Biopsy neutrophilia, neutrophil chemokine and receptor gene expression in severe exacerbations of chronic obstructive pulmonary disease.** *Am J Respir Crit Care Med* 2003, **168:**968-975.

36. Saetta M, Di Stefano A, Maestrelli P, Turato G, Ruggieri MP, Roggeri A, Calcagni P, Mapp CE, Ciaccia A, Fabbri LM: **Airway eosinophilia in chronic bronchitis during exacerbations.** *Am J Respir Crit Care Med* 1994, **150:**1646-1652.

37. Zhu J, Qiu YS, Majumdar S, Gamble E, Matin D, Turato G, Fabbri LM, Barnes N, Saetta M, Jeffery PK: **Exacerbations of Bronchitis: bronchial eosinophilia and gene expression for interleukin-4, interleukin-5, and eosinophil chemoattractants.** *Am J Respir Crit Care Med* 2001, **164:**109-116.

38. Saetta M, Di Stefano A, Maestrelli P, Turato G, Mapp CE, Pieno M, Zanguochi G, Del Prete G, Fabbri LM: **Airway eosinophilia and expression of interleukin-5 protein in asthma and in exacerbations of chronic bronchitis.** *Clin Exp Allergy* 1996, **26:**766-774.
